# Neighborhood frequency effects in simple and complex span: Do high-frequency neighbors help or hurt?

**DOI:** 10.3758/s13421-024-01658-w

**Published:** 2024-11-05

**Authors:** Molly B. MacMillan, Ian Neath, Steven Roodenrys

**Affiliations:** 1 Memorial University of Newfoundland, St. John’s, NL Canada; 2 Department of Psychology, Virginia Tech, 390 Drillfield Drive, Blacksburg, VA 24060 USA; 3 University of Wollongong, Wollongong, NSW Australia

**Keywords:** Orthographic neighborhood, Phonological neighborhood, Neighborhood frequency, Serial recall, Complex span

## Abstract

A word's orthographic neighborhood is the set of words that differ from the target word by one letter. Both Roodenrys (2009) and Robert et al. (*Journal of Psycholinguistic Research, 44,* 119-125, 2015) posit that orthographic neighbors are activated when the target word is encountered in tasks such as simple and complex span. The two accounts differ in that the former predicts a beneficial effect of this activation, because it produces feedback activation that helps redintegrate the target word, whereas the latter predicts a detrimental effect, because the need to overcome the greater interference from the larger number of higher-frequency items reduces the processing resources available. Four experiments assess the predictions of these two accounts. Experiments 1 and 2 found a beneficial effect of having a higher- compared with a lower-frequency neighborhood in both a simple and a complex span task. Experiments 3 and 4 found no detrimental effect of having one or more neighbors with higher frequency than the target in both a simple and complex span task. Implications for the two theories are discussed.

**Supplementary information:**

The online version contains supplementary material available at 10.3758/s13421-024-01658-w.

## Introduction

In 1887, Jacobs defined the term *span* as the highest number of words that could be correctly reproduced immediately. Researchers quickly established that this simple span measure was affected by various lexical and long-term memory factors (for an early review, see Blankenship, 1938). Following the formulation of Baddeley and Hitch's (1974) working memory, complex span tasks were developed to assess the capacity of working memory. Whereas simple span tasks, such as that of Jacobs, emphasize storage, complex span tasks were designed to also require "the simultaneous processing of additional information” (Conway et al., 2005, p. 771). In this paper, we assess two competing predictions of whether orthographic neighborhood frequency affects performance on these two tasks.

One widely used definition of an orthographic neighbor is a word that differs from the target by a single letter (Coltheart et al., 1977). For example, orthographic neighbors of *cat* include *bat*, *cot*, and *cap*. Other definitions allow for the addition or subtraction of letters. For example, Yarkoni et al. (2008) proposed a measure called *orthographic Levenshtein distance* (OLD), which is based on the number of edits required to transform one word to another. A word's orthographic neighborhood is the set of these neighbors. On tests of immediate serial recall, lists of words with a large orthographic neighborhood are better recalled than otherwise comparable lists of words with a small neighborhood (e.g., Allen & Hulme, 2006; Guitard et al., 2024a).^1^

One explanation of this beneficial effect of neighborhood size is Roodenrys’ (2009) redintegration framework. According to this account, the degraded list items can serve as input to an interactive activation network. The orthographic neighbors of each list item are partially activated, so words with more neighbors will partially activate more items than words with fewer neighbors. The activation from the neighbors feeds back to the list item and so items with more neighbors receive more feedback than items with fewer neighbors, which aids with subsequent redintegration.

In addition to examining the size of an orthographic neighborhood, one can also look at the frequency of the words that comprise the neighborhood. One can compute the mean frequency of the orthographic neighborhood as a whole and distinguish between higher-frequency neighborhoods and lower-frequency neighborhoods. One can also look at individual orthographic neighbors and assess their frequency relative to the target. For example, according to the Brysbaert and New (2009) norms, *cap*, an orthographic neighbor of *cat*, has a lower frequency than *cat*, but *can*, another orthographic neighbor of *cat*, has a higher frequency than *cat*.

Although initially expressed in terms of number of neighbors, the redintegration account can also accommodate the effects of the frequency of the neighbors given the assumption that higher-frequency neighbors receive more partial activation than lower-frequency neighbors. Other things being equal, there should be more activation feedback from words with higher-frequency neighborhoods than words with lower-frequency neighborhoods, and more feedback aids redintegration.

However, Robert et al. (2015) reported results that are potentially problematic for this view. Rather than manipulating the frequency of the orthographic neighborhood, they manipulated whether the to-be-remembered words had any neighbors that were higher in frequency than the target word. In the *none* condition, all of the orthographic neighbors were of lower frequency than the target word. In the *some* condition, each target word had at least one orthographic neighbor that was of higher frequency than the target. Subjects heard two to five sentences and then were asked to recall the last word in each sentence. When subjects were shown a series of two to four sentences, there was no difference in performance between the *some* and the *none* conditions. However, when subjects were shown a series of five sentences, recall performance was significantly better for words with no higher-frequency neighbors than for words that had some higher-frequency neighbors.

Robert et al. (2015) suggested that having neighbors that are higher in frequency than the target reduces recall because it leads to more interference. Specifically, like Roodenrys (2009), they assumed that orthographic neighbors are co-activated. However, whereas Roodenrys posited that this activation of neighbors would benefit the target word because it produces more feedback activation that converges on the target word, Robert et al. suggested that this activation of neighbors would harm the target word because the need to overcome the greater interference from the larger number of higher-frequency items would reduce the processing resources available. Consistent with this view, they noted that the detrimental effect of having orthographic neighbors that were higher in frequency than the target was seen only on the hardest complex span task where processing resources would be most taxed.

The work showing a beneficial effect of orthographic neighborhood frequency is not necessarily incompatible with the work showing a detrimental effect of having orthographic neighbors that are higher in frequency than the target word. However, the two explanations do seem somewhat at odds. One set of results has led to the suggestion, following Roodenrys (2009), that the co-activation of neighbors helps recall because their activation feeds back to the target. Specifically, to the extent that higher-frequency words have more activation, this view predicts a beneficial effect of having a higher-frequency neighborhood. In contrast, the other set of results has been interpreted as showing that the co-activation of neighbors hurts recall because their activation reduces processing resources (Robert et al., 2015). Specifically, if the orthographic neighborhood contains words that are higher in frequency than the target, recall of the target will be worse: Higher-frequency words have more activation and this leads to a depletion of processing resources, which in turn interferes with recall. The four experiments reported here were designed to explore these different accounts.

Experiments 1 and 2 manipulated orthographic neighborhood frequency, the mean frequency of all orthographic neighbors of the target word. On the assumption that higher-frequency words are more activated than lower-frequency words, Roodenrys’ (2009) account predicts a memory advantage for words with higher-frequency neighborhoods because there should be more activation feedback to the target, which helps with redintegration. In contrast, the impairment account of Robert et al. (2015) predicts the opposite on the grounds that higher-frequency neighbors take up more processing resources. The two experiments differ only in the type of test, immediate serial recall (Experiment 1) and operation span (Experiment 2).

Experiments 3 and 4 controlled for orthographic neighborhood frequency but manipulated whether the target word had any orthographic neighbors that were higher in frequency than the target, the same manipulation as Robert et al. (2015). Half of the lists used words that had at least one orthographic neighbor of higher frequency than the target word whereas the other half used words with no higher-frequency neighbors. Because we controlled for both the number of orthographic neighbors as well as the mean frequency of the orthographic neighborhood, Roodenrys’ account predicts no difference in memory performance: It does not matter that in the *some* condition, some of the orthographic neighbors are higher in frequency than the target. What determines performance is the mean frequency of the orthographic neighborhood as a whole and given these are equated, words in both conditions will receive similar levels of feedback activation. In contrast, the Robert et al. account predicts worse memory for words with higher-frequency neighbors because the activation of these words will reduce available processing resources.

## Experiment 1

The purpose of Experiment 1 was to determine whether a beneficial orthographic neighborhood frequency effect could be observed with standard serial recall when the size of the neighborhood is held constant.

### Ethics

The research was approved by Memorial University's Interdisciplinary Committee on Ethics in Human Research.

### Sample size

We used Bayes factor design analysis (BFDA; Schönbrodt & Wagenmakers, 2018) to estimate a sample size that would be likely to provide informative Bayes factors and unlikely to result in inconclusive Bayes factors using the BFDA package (Schönbrodt & Stefan, 2019). The two most critical statistical tests will be within-subjects Bayesian *t*-tests comparing recall of two types of words at the longest-length complex span task (i.e., high and low neighborhood frequency words in Experiment 2, and words with some or no higher-frequency neighbors in Experiment 4). We used an effect size of *d* = 0.5 for the alternative hypothesis and an effect size of *d* = 0.0 for the null hypothesis. The decision boundary was set at BF > 3.0 for each hypothesis, and 10,000 simulations were conducted using a non-directional Bayesian within-subjects *t* test with default priors. For the alternative hypothesis, the simulations indicated that with 50 subjects 82.2% of the samples indicated evidence for the alternative hypothesis (BF > 3), only 16.6% were inconclusive (0.333 < BF < 3), and 1.2% indicated evidence for the null hypothesis (BF < 0.333). For the null hypothesis, simulations indicated that 80.0% of the samples showed evidence for the null hypothesis (BF < 0.333); only 18.8% were inconclusive (0.333 < BF < 3), and 1.2% showed evidence for the alternative hypothesis (BF > 3). A sample size of 50, then, should be able to detect evidence for either an effect of neighborhood frequency or a null result, and should be unlikely to result in inconclusive evidence.

### Subjects

Fifty volunteers from Prolific were paid £8.00 per hour (prorated) for their participation. The inclusion criteria were: (1) native speaker of English; (2) age between 19 and 39 years; and (3) at least a 90% approval rating on prior participation. The mean age was 27.64 (*SD* = 6.11, range 19–39) years and 32 self-identified as female and 18 as male.

### Stimuli

The stimuli were 75 words that had high-frequency neighbors and 75 words that had low-frequency neighbors. These nouns were equated on a number of dimensions including number of orthographic neighbors (Coltheart et al., 1977) and both orthographic and phonological Levenshtein distance (Yarkoni et al., 2008). There was no overlap in the frequency of the neighbors on any of three measures: (1) CELEX frequency of orthographic neighbors, low = 0.24–25.89 and high = 30.88–1,486.19; (2) frequency of neighbors based on orthographic Levenshtein distance, 6.71–8.59 versus 8.60–10.16; and (3) frequency of neighbors based on phonological Levenshtein distance, 7.10–8.64 versus 8.65–10.66; see the Appendix in the Online Supplemental Material for details. We note, however, that we could not equate the words for the number of neighbors that were higher in frequency than the target word. The low neighborhood frequency words had a mean of 2.85 higher-frequency neighbors compared to 5.32 for the high neighborhood frequency words, *t*(148) = 4.761, *p* < 0.001. According to the processing resource reduction account, this should work against recall of the high neighborhood frequency words and therefore against finding a neighborhood frequency effect.

### Procedure

After consenting to participate, the subjects were reminded of the instructions. They saw a list of six words, shown one at a time for 1 s in the center of the display in 28-point Helvetica font. They were asked to read these words silently. Immediately after the last item disappeared, the subjects were prompted to type in the first word, then the second word, and so on. Subjects were encouraged to guess or they could click on a button labelled “skip.” There was no time limit on the recall period. After six responses had been made, the subject could click on a “Start Next Trial” button when ready.

There were 20 trials, half with low and half with high neighborhood frequency words. For each subject, the words for the up-coming trial were randomly selected without replacement from the appropriate pool and then randomly ordered. The order of the trials was randomly determined for each subject.

### Data analysis

The data were analyzed using JASP (JASP Team, 2023). For the Bayesian repeated-measures ANOVA, we report either a Bayes factor, BF_10_, that indicates evidence for the alternative hypothesis or a Bayes factor, BF_01_, that indicates evidence for the null hypothesis. We interpret a value between 3 and 10 as indicating substantial evidence; a value between 10 and 30 indicating strong evidence; a value between 30 and 100 indicating very strong evidence; and a value greater than 100 indicating decisive evidence (Wetzels et al., 2011). Main-effect models were evaluated with respect to a random-effects error model, and interaction models were evaluated with respect to a main-effects model.

### Results and discussion

**Fig. 1:**
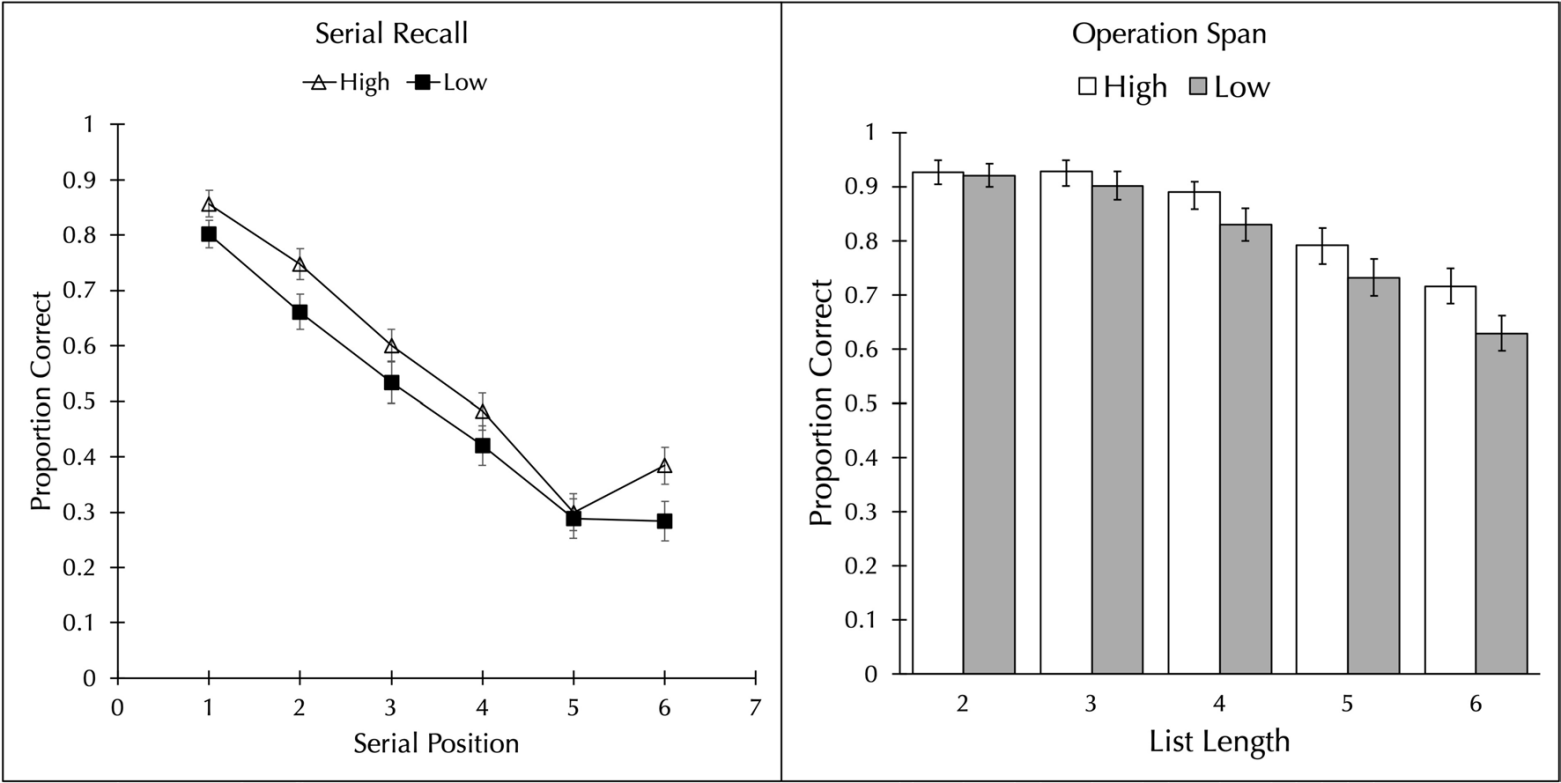
Proportion of words correctly recalled as a function of whether the word has a high- or low-frequency neighborhood in Experiment 1, when the task was serial recall (**left panel**), and Experiment 2, when the task was operation span (**right panel**). Error bars show the standard error of the mean

The proportion of words correctly recalled in order was analyzed by a 2 word type (high vs. low neighborhood frequency) × 6 serial position repeated-measures Bayesian ANOVA.^2^ As can be seen in the left panel of Fig. 1, the proportion of high neighborhood frequency words recalled (*M* = 0.575, *SD* = 0.161) was greater than the proportion of low neighborhood frequency words recalled (*M* = 0.511, *SD* = 0.185), BF_10_ = 9.616, *d* = 0.545. There was the usual effect of serial position, BF_10_ = 1.47 × 10^72^, and no interaction, BF_01_ = 11.785.

The pattern of results was the same when the data were scored without regard to order; that is, a word was counted as correctly recalled regardless of the position in which it was recalled. The proportion of high neighborhood frequency words recalled (*M* = 0.660, *SD* = 0.138) was greater than the proportion of low neighborhood frequency words recalled (*M* = 0.593, *SD* = 0.165), BF_10_ = 191.376, *d* = 0.622. There was the usual effect of serial position, BF_10_ = 1.79 × 10^60^ and no interaction, BF_01_ = 61.455.

Words with high neighborhood frequency were better recalled on an immediate serial recall test than words with low neighborhood frequency. The results are consistent with the prediction of Roodenrys' (2009) redintegration framework that orthographic neighbors are partially activated by the list items and feedback from these neighbors can facilitate subsequent redintegration. If the mean frequency of the neighborhood is higher, there is more partial activation of the neighbors and thus more feedback activation, which benefits redintegration. The results are seemingly incompatible with the view that high frequency neighbors deplete processing resources and thereby have a detrimental effect on recall. However, it may be the case that a more demanding task is needed. Therefore, Experiment 2 used operation span, a common complex span task, rather the immediate serial recall because complex span tasks are generally seen as requiring more processing resources than simple span tasks (e.g., Conway et al., 2005).

## Experiment 2

The purpose of Experiment 2 was to determine whether the orthographic neighborhood frequency effect observed with serial recall in Experiment 1 would also be observed in a complex span task. We used an operation span task rather than the sentence span task used by Robert et al. (2015) for two reasons. First, performance on the math task can be easily measured to ensure that subjects are not just focusing on the words, and second, the only words processed in an operation span task (other than mathematical terms) are the to-be-remembered words, removing a potential source of interference.

### Subjects

Fifty different volunteers from Prolific were paid £8.00 per hour (prorated) for their participation. The mean age was 27.64 (*SD* = 5.71, range 19–38) years and 38 subjects self-identified as female and 12 as male.

### Stimuli

The stimuli were the same as in Experiment 1.

### Procedure

After consenting to participate, the subjects were reminded of the instructions. They first saw a simple math question, such as “Is (6 ÷ 2) + 1 = 4?” which they were asked to read out loud (i.e., “Is six divided by two plus one equal to four”) and then click on one of two buttons labelled “Yes” and “No” to indicate the answer. They then saw a word. Math problems alternated with words until the desired list length had been reached. Then, the same serial recall instructions as in Experiment 1 were given.

The first two trials were practice and had a list length of 2. One trial had low neighborhood frequency words and the other had high neighborhood frequency words. These trials were not included in the analysis. There then followed 20 experimental trials. There were four trials each of list length 2, 3, 4, 5, and 6. Of the four trials at each list length, half had low neighborhood frequency words and half had high neighborhood frequency words. The words used on each trial and the order of the 20 experimental trials were randomly determined for each subject.

### Results and discussion

There was no difference in the proportion of math questions answered correctly on high neighborhood frequency trials, 0.918 (*SD* = 0.050), compared to low neighborhood frequency trials, 0.920 (*SD* = 0.047), BF_01_ = 5.952, *d* = 0.061.

Memory performance was measured in three ways. The OSPAN measure is defined as the sum of list lengths correctly recalled. For example, if all five words from a list length five trial are correctly recalled in order, then five is added to the OSPAN score. If four words from a list length five trial are correctly recalled in order, but one word is not recalled, then nothing is added to the OSPAN score. OSPAN was higher for high neighborhood frequency trials than low neighborhood frequency trials, 21.86 (SD = 10.09) versus 19.42 (SD = 9.39), BF_10_ = 7.109, *d* = 0.418.^3^

The proportion of words correctly recalled in order was analyzed by a 2 neighborhood frequency × 5 list length repeated measures Bayesian ANOVA. As can be seen in the right panel of Fig. 1, more words were correctly recalled from high neighborhood frequency trials (*M* = 0.843, *SD* = 0.118) than low neighborhood frequency trials (*M* = 0.795, *SD* = 0.148), BF_10_ = 8.711, *d* = 0.617. There was a main effect of list length, BF_10_ = 3.21 × 10^22^, with performance decreasing as the list length increased, but no interaction, BF_01_ = 15.937. When just the data from list length six are considered, there is still a difference: The proportion of words correctly recalled on high neighborhood frequency trials (*M* = 0.713, *SD* = 0.232) was higher than for low neighborhood frequency trials (*M* = 0.622, *SD* = 0.221), BF_10_ = 8.000, *d* = 0.425.

The pattern of results was the same when the data were scored without regard to order: More words were correctly recalled from high neighborhood frequency trials (*M* = 0.877, *SD* = 0.097) than low neighborhood frequency trials (*M* = 0.838, *SD* = 0.117), BF_10_ = 6.181, *d* = 0.572. There was a main effect of list length, BF_10_ = 1.59 × 10^18^, with performance decreasing as the list length increased, but no interaction, BF_01_ = 6.209. When just the data from list length six are considered, there is still a difference: The proportion of words correctly recalled on high neighborhood frequency trials (*M* = 0.778, *SD* = 0.192) was higher than for low neighborhood frequency trials (*M* = 0.690, *SD* = 0.205), BF_10_ = 44.687, *d* = 0.518.

Experiment 2 found the same results as Experiment 1 despite the change to a more demanding complex span task: Words with high neighborhood frequency were better recalled than words with low neighborhood frequency. As with Experiment 1, the results are consistent with Roodenrys’ (2009) redintegration account and appear problematic for the processing resource account of Robert et al. (2015).

Even though the results of Experiments 1 and 2 confirm a strong prediction of the interactive activation account of Roodenrys (2009), it might be objected that it is not the most appropriate test of the resource depletion account of Robert et al. (2015). The reason is that the latter researchers focused on whether the orthographic neighborhood contained some words that were higher in frequency than the target (the *some*) condition or contained no words that were higher in frequency than the target (the *none*) condition. If this manipulation is done while holding orthographic neighborhood size and frequency constant, the two accounts make different predictions. Because neighborhood size and neighborhood frequency are controlled, Roodenrys’ (2009) account predicts no difference. On average, both the *some* and *none* lists will receive the same amount of feedback activation because both are equated for neighborhood frequency. In contrast, the resource depletion account of Robert et al. (2015) predicts worse performance for the *some* condition because the presence of orthographic neighbors that are higher in frequency than the target will reduce processing resources. Therefore, Experiments 3 and 4 parallel Experiments 1 and 2 except they compare recall of words that have some orthographic neighbors that are higher in frequency than the target word (called the *some* condition) to words that have no orthographic neighbors that are higher in frequency than the target word (called the *none* condition).

## Experiment 3

A new set of stimuli were created that differed in the same way as those used by Robert et al. (2015). In the *some* condition, each target word had at least one orthographic neighbor that was higher in frequency than the target word whereas in the *none* condition, no target word had any orthographic neighbors that were higher in frequency. For both types of list, the size and frequency of the orthographic neighborhoods was equated.

### Subjects

Fifty different volunteers from Prolific participated. The mean age was 27.68 (*SD* = 6.21, range 19–39) years and 29 subjects self-identified as female and 21 as male.

### Stimuli

The stimuli were 63 words that had at least one higher-frequency orthographic neighbor (*M* = 1.52) and 63 words that did not have any higher-frequency orthographic neighbors (*M* = 0.00), as defined by Coltheart et al. (1977). The two sets of words were equated on a number of dimensions including number of orthographic neighbors (Coltheart’s N) and both orthographic and phonological Levenshtein distance and frequency (Yarkoni et al., 2008); see the Appendix (Online Supplemental Material) for details.

### Procedure

The procedure was identical to that of Experiment 1.

### Results and discussion

**Fig. 2:**
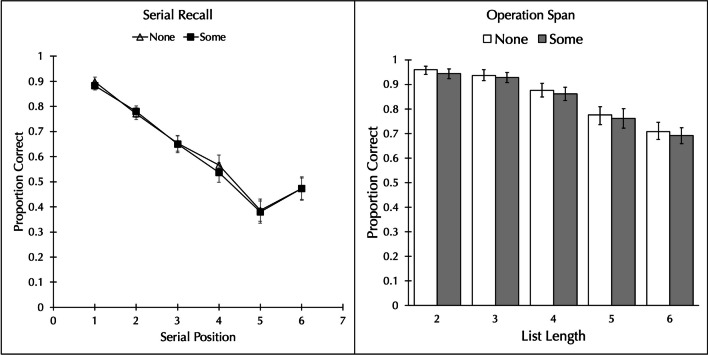
Proportion of words correctly recalled as a function of whether the word has no neighbors with a higher frequency (none) or has neighbors with higher frequency (some) in Experiment 3, when the task was serial recall (**left panel**), and Experiment 4, when the task was operation span (**right panel**). Error bars show the standard error of the mean

The proportion of words correctly recalled in order was analyzed by a 2 word type (no vs. some higher-frequency neighbors) × 6 serial position repeated-measures Bayesian ANOVA.^4^ As can be seen in the left panel of Fig. 2, there was no effect of whether a word had orthographic neighbors that were higher in frequency or not. The proportion of words correctly recalled in the *none* condition (*M* = 0.638, *SD* = 0.173) did not differ from that of the *some* condition (*M* = 0.632, *SD* = 0.171), BF_01_ = 7.246, *d* = 0.076. There was the usual effect of serial position, BF_10_ = 1.91 × 10^49^ and no interaction, BF_01_ = 89.906.

The pattern of results was the same when the data were scored without regard to order: there was no effect of whether a word had orthographic neighbors that were higher in frequency or not. The proportion of words correctly recalled in the *none* condition (*M* = 0.715, *SD* = 0.135) did not differ from that of the *some* condition (*M* = 0.702, *SD* = 0.147), BF_01_ = 5.102, *d* = 0.198. There was the usual effect of serial position, BF_10_ = 1.18 × 10^36^ and no interaction, BF_01_ = 21.549.

Experiment 3 found no evidence that whether a word has or does not have an orthographic neighbor that is higher in frequency affects performance on immediate serial recall. However, it might be objected that the detrimental effect observed by Robert et al. (2015) appeared only under the most demanding condition, a list length five of a complex span task. Therefore, Experiment 4 changed to a complex span task and the longest list length was six. Whereas a detrimental effect of having higher-frequency neighbors may not be apparent on the shorter list lengths, it should appear on list lengths five and six.

## Experiment 4

Experiment 3 found no evidence that serial recall is affected by whether a word has an orthographic neighbor that is higher in frequency than the target word. This null result differs from that of Robert et al. (2015) but one prominent difference is that Experiment 3 used a simple span task whereas Robert et al. used a complex span task. The purpose of Experiment 4, then, was to use the same stimuli as in Experiment 3 but with a complex span task. As in Experiment 2, we used an operation span task rather than the word span task used by Robert et al. (2015) to minimize interference: In the former task, the only words (other than mathematical terms) are the to-be-remembered items.

### Subjects

Fifty different volunteers from Prolific were paid £8.00 per hour (prorated) for their participation. The mean age was 29.72 (*SD* = 6.24, range 20–39) years and 33 subjects self-identified as female, 16 self-identified as male, and one self-identified as other.

### Stimuli

The stimuli were the same as in Experiment 3.

### Procedure

The procedure was the same as in Experiment 2.

### Results

There was no difference in the proportion of math questions answered correctly on trials with words from the *none* condition, 0.938 (*SD* = 0.045), compared to trials with words from the *some* condition, 0.934 (*SD* = 0.046), BF_01_ = 5.556, *d* = 0.082.

We again score the data in three ways. OSPAN did not differ, with mean OSPAN of 23.12 (*SD* = 8.38) for words in the *none* condition compared to 22.46 (*SD* = 8.84) for words in the *some* condition, BF_01_ = 4.926, *d* = 0.173.^5^

The proportion of words correctly recalled was analyzed by a 2 word type (none vs. some higher-frequency neighbors) × 5 list length repeated-measures Bayesian ANOVA. As can be seen in the right panel of Fig. 2, there was no difference in recall between the none and some conditions. The proportion of words correctly recalled in the *none* condition (*M* = 0.852, *SD* = 0.119) did not differ from the *some* condition (*M* = 0.839, *SD* = 0.126), BF_01_ = 5.208, *d* = 0.173. There was a main effect of list length, BF_10_ = 4.29 × 10^19^, with performance decreasing as the list length increased, but no interaction, BF_01_ = 91.974. When just the data from list length six are considered, there is still no difference: The proportion of words correctly recalled in the none condition (*M* = 0.713, *SD* = 0.232) did not differ from the some condition (*M* = 0.700, *SD* = 0.204), BF_01_ = 5.967, *d* = 0.060.

The pattern of results was the same when the data were scored without regard to order: there was no difference in recall between the *none* and *some* conditions. The proportion of words correctly recalled in the *none* condition (*M* = 0.887, *SD* = 0.090) did not differ from the *some* condition (*M* = 0.874, *SD* = 0.111), BF_01_ = 4.255, *d* = 0.186. There was a main effect of list length, BF_10_ = 1.50 × 10^18^, with performance decreasing as the list length increased, but no interaction, BF_01_ = 20.306. When just the data from list length six are considered, there is still no difference: The proportion of words correctly recalled in the *none* condition (*M* = 0.792, *SD* = 0.159) did not differ from the *some* condition (*M* = 0.777, *SD* = 0.174), BF_01_ = 5.164, *d* = 0.099.

## General discussion

Both Roodenrys (2009) and Robert et al. (2015) posit that orthographic neighbors of a word are co-activated and both also assume that higher-frequency neighbors produce more feedback activation than lower-frequency neighbors. However, they differ in the consequence of this activation. In Roodenrys' account, this activation of neighbors will benefit the target word because each neighbor will produce feedback activation that converges on the target word and thereby enhances redintegration. In Robert et al.'s account, on the other hand, this activation of neighbors will harm the target word because the larger number of higher-frequency items will reduce the availability of processing resources. We evaluated the predictions of these two accounts using two different sets of stimuli and two different manipulations.

In Experiments 1 (serial recall) and 2 (operation span), the stimuli differed in orthographic neighborhood frequency but were equated for number of orthographic neighbors. In both experiments, the words with higher-frequency neighborhoods were better recalled than words with lower-frequency neighborhoods. According to Roodenrys' (2009) view, this is because the neighbors were co-activated and the feedback from these activated neighbors boosted the target item. On the assumption that higher-frequency neighbors are more activated, there should be a benefit for words with higher-frequency neighborhoods compared to those with lower-frequency neighborhoods. According to Robert et al. (2015), the more activated the neighbors, the larger the reduction in available processing resources. Therefore, words with higher-frequency neighborhoods are predicted to be recalled worse than words with low-frequency neighborhoods.

In Experiments 3 (serial recall) and 4 (operation span), different stimuli were used. In the *some* condition, the words had some orthographic neighbors that were higher in frequency than the target whereas in the *none* condition, the words had no orthographic neighbors that were higher in frequency than the target. There was no difference in recall as a function of whether a word did or did not have neighbors that were higher in frequency than the target. According to Roodenrys' (2009) account, there is no difference in recall because the two sets of words were equated for neighborhood frequency and therefore, on average, the words in both conditions received the same assistance from activation feedback. According to Robert et al. (2015), the words in the *some* condition should have been recalled worse than those in the *none* condition because the presence of neighbors that were of higher frequency than the target should have reduced the availability of processing resources.

There are a number of potential explanations for why our results, particularly those of Experiment 4, differed from those reported by Robert et al. (2015). First, Robert et al. used a sentence span task in which interference from the many non-target words might play a role. In contrast, Experiment 4 used operation span to minimize the possibility of interference from other words. Although there were some additional words in the operation span task – the subjects were asked to read the mathematical operation out loud – these extra words were easily distinguishable from the to-be-remembered words (e.g., they were numbers or mathematical terms) and should not have interfered. In the sentence span task, it may be that it is the presence of the other words that reduced processing capacity and drove the effect rather than the nature of the to-be-remembered items.

A second possibility is that the operation span task we used was not as demanding as the sentence span task Robert et al. (2015) used. Robert et al. noted that a reduction in processing resources will affect memory only when the task is sufficiently demanding and in the shorter list lengths in their own data they found no difference, the same result we found at all list lengths. Although possible, we think this unlikely for two reasons. First, the overall performance level on the longest lists in Experiment 4 was similar to that of Robert et al. (2015), suggesting the two tasks were roughly equally difficult. Second, we used a list-length of six, one item more than Robert et al.'s maximum list length of five.

A third possibility has to do with the stimuli used. First, Robert et al. (2015) equated the words in the *some* and *none* conditions on only a few dimensions, including length (number of phonemes, number of letters, and number of syllables) and frequency. As Table A2 (Online Supplemental Material) shows, we equated our stimuli on many more dimensions. It is possible that the words used by Robert et al. differed on some dimension that we controlled for and it is that difference between conditions that drove their results. A second difference in the stimuli is that Robert et al. had a slightly larger manipulation than we did; in other words, our *some* condition had fewer words that were higher in frequency than the target. It may be the case that with a larger manipulation we would have seen an effect. Finally, the stimuli in the two studies may have differed in how the orthographic neighbors deviate from the target word.^6^ For example, in visual word recognition, orthographic neighbors that differ from a target by an interior letter are more interfering than neighbors that differ by an initial or final letter (see, e.g., Perea, 1998). It may be the case that the same applies to memory, in that neighbors that differ by an interior letter are less disruptive than those that differ by an initial or end letter. The suggestion, then, is that our stimuli may have had more of former whereas the stimuli of Robert et al. (2015) had more of the latter. Because of these differences, future researchers should ensure that they equate their conditions on at least as many dimensions as we did, have a manipulation at least as large as that of Robert et al., and ensure that the orthographic neighbors in the *some* and *none* conditions have the same number of words differing by initial, interior, and final letters. However, we note that even if these were factors, they would not explain why the results of Experiments 1 and 2 are contrary to the prediction of the resource depletion account.

A fourth possibility may be due to sample size differences.^7^ We used the Bayes Factor design analysis package (Schönbrodt & Stefan, 2019) to estimate a sample size that is likely to detect evidence for either an effect or a null result and unlikely to result in an inconclusive Bayes factor. Our sample size of 50 is double that of Robert et al. (2015). Their design was thus less powerful, and indeed, their main effect of neighborhood frequency was not significant (*p* = 0.21).

A final possibility may be due to language. Robert et al. (2015) used French words and French-speaking subjects whereas we used English words and English-speaking subjects. Whereas feedback from high-frequency neighbors is beneficial in the English working memory system, it may be detrimental in non-English working memory systems. This idea is consistent with results from the psycholinguistic literature concerning neighborhood frequency. Andrews (1997) provided an extensive review which revealed that on a number of tasks such as lexical decision, neighborhood frequency had different effects for English on the one hand and languages such as French and Spanish on the other. For the latter, there was a consistent finding of inhibitory effects which was absent when English was used. One possible explanation for these language-based differences was suggested by Sears et al. (2006). They noted that English words are, on average, shorter than words in languages such as French and Spanish. Short words have more orthographic neighbors than long words, and short words are generally of higher frequency than longer words. They noted that as a result, English words will, on average, have more higher-frequency neighbors. “This neighborhood structure for English words (i.e., larger neighborhoods and many higher-frequency neighbors) may necessitate a lexical processor with weaker inhibitory connections than those in other languages” (Sears et al., 2006, p. 1059). While not all variables that affect psycholinguistic tasks affect memory tasks in the same way, the presence of neighborhood frequency differences as a function of language in lexical decision tasks suggest it is not implausible that similar differences might be observable in memory tasks. For example, if the lexical processor does differ as a function of language, it would resolve the discrepancy between the results of the current paper and those of Robert et al. (2015).

Regardless of the reason for why the current results differ from those of Robert et al. (2015), they add to the large body of previous work showing that working memory performance is always contaminated by long-term memory/linguistic factors, such as whether an item is a word or a nonword (Hulme et al., 1991), is abstract or concrete (Walker & Hulme, 1999), is of high or low frequency (Roodenrys & Quinlan, 2000), is semantically related or unrelated (Neath et al., 2022), or has a large or small orthographic (Allen & Hulme, 2006) or phonological (Roodenrys et al., 2002) neighborhood, to name only a few such factors.

## Summary

The processing reduction account (Robert et al., 2015) predicts that having higher-frequency orthographic neighbors will impair recall whereas the redintegration account (Roodenrys, 2009) predicts that having higher-frequency orthographic neighbors will facilitate recall. In Experiments 1 and 2, higher-frequency neighborhoods were associated with better recall, not worse, in both a serial recall and complex span task. In Experiments 3 and 4, there was no difference in recall of words that had some higher-frequency neighbors compared to those that had no higher-frequency neighbors when the conditions were equated for neighborhood size and frequency. This pattern of results is consistent with the redintegration account and poses a problem for the processing reduction account.

## Supplementary information

Below is the link to the electronic supplementary material.

Supplementary file1 (DOCX 37.1 KB)

## Data Availability

The raw data and stimuli are available via the Open Science Framework at 10.17605/OSF.IO/C2B7W

## Footnotes

1 This neighborhood size effect is also observed on other memory tests, including simple span (Roodenrys et al., 2002), reconstruction of order (Jalbert et al., 2011a, b), and serial recognition (Guitard et al., 2024b).

2 The responses were first checked for spelling and typing errors. Out of 3,000 responses, 16 high neighborhood frequency words were flagged as misspelled compared to 28 low neighborhood frequency words. Correcting these words changed seven high frequency responses to correct and 13 low frequency responses to correct. Because the statistical results do not change, we report only analyses of the uncorrected data.

3 The responses were first checked for spelling and typing errors. Out of 4,000 responses, ten high neighborhood frequency words were flagged as misspelled compared to 14 low neighborhood frequency words. Correcting these words changed six high neighborhood frequency responses to correct and eight low neighborhood frequency responses to correct. Because the statistical results do not change, we report only analyses of the uncorrected data.

4 The responses were first checked for spelling and typing errors. Out of 3,000 responses, 26 of the words in the *none* condition were flagged as misspelled compared to 31 words in the *some* condition. Correcting these words changed 16 and 14 responses to correct, respectively. Because the statistical results do not change, we report only analyses of the uncorrected data.

5 The responses were first checked for spelling and typing errors. Out of 4,000 responses, ten words in the *none* condition were flagged as misspelled compared to 18 words in the *some* condition. Correcting these words changed seven responses to correct for the former and eight responses to correct for the latter. Because the statistical results do not change, we report only analyses of the uncorrected data.

6 We thank an anonymous reviewer for suggesting this possibility.

7 We thank an anonymous reviewer for suggesting this.
