# Supplementary material for: Neighborhood frequency effects in simple and complex span: Do high-frequency neighbors help or hurt?
Source: Mem Cognit. 2024 Nov 5;52(8):1871–81. doi: 10.3758/s13421-024-01658-w (PMC11588882; doi:10.3758/s13421-024-01658-w)
Supplement: Supplementary file 1 — Supplementary file1 (DOCX 37.1 KB) [file 13421_2024_1658_MOESM1_ESM.docx]

Appendix

Note: CELEX: Log base 10 CELEX frequency; Orth: number of orthographic neighbors (Coltheart’s *N*); OrthZ: a z score based on Orth [see Storkel, 2004]; OrthF: frequency of orthographic neighbors; C2: constrained bigram frequency; C2Z: a z score based on C2; U2: constrained bigram frequency; U2Z: a z score based on U2; NHFN: Number of higher frequency neighbors (from Medler & Binder, 2003); LgWF: log base 10 SUBTLEX_US_ frequency; LgCD: log base 10 SUBTLEX_US_ contextual diversity (from Brysbaert & New, 2009); zipf UK: zipf frequency SUBTLEX_UK_; zipf BNC: zipf British National Corpus frequency (from van Heuven et al., 2014); LgHAL: log base 10 HAL frequency; OLD: orthographic Levenshtein distance; OLDF: frequency of the orthographic Levenshtein neighbors; PLD: phonological Levenshtein distance; PLDF: frequency of the phonological Levenshtein neighbors; NPhon: number of phonemes; NSyll: number of syllables; NLet: number of letters (from Balota et al., 2007); AoA: tested age of acquisition (from Brysbaert & Biemiller, 2017); Cnc.M: mean concreteness; Known: proportion of respondents indicating they knew the word (from Brysbaert et al., 2014); and WNPL: WordNet path length, a measure of semantic relatedness (from Ensor et al., 2021). ***Bold italic*** indicates a significant difference.

Table A1: Descriptive properties of the stimuli used in Experiments 1 and 2.

|  | Low NF | | High NF | |  |  |
| --- | --- | --- | --- | --- | --- | --- |
|  | *M* | *SD* | *M* | *SD* | *t* | *p* |
| CELEX | 4.10 | 0.64 | 4.14 | 0.73 | 0.38 | 0.71 |
| Orth | 10.23 | 5.45 | 10.77 | 5.55 | 0.61 | 0.54 |
| OrthZ | 0.22 | 0.74 | 0.27 | 0.77 | 0.37 | 0.71 |
| ***OrthF*** | ***12.35*** | ***6.75*** | ***173.60*** | ***202.98*** | ***6.88*** | ***< 0.01*** |
| C2 | 76.15 | 48.63 | 70.83 | 47.61 | 0.68 | 0.50 |
| C2Z | 0.23 | 0.72 | 0.35 | 0.82 | 0.98 | 0.33 |
| U2 | 6825.67 | 4422.46 | 7991.15 | 4774.60 | 1.55 | 0.12 |
| ***U2Z*** | ***-0.14*** | ***0.81*** | ***0.22*** | ***1.01*** | ***2.43*** | ***0.02*** |
| ***NHFN*** | ***2.85*** | ***2.85*** | ***5.32*** | ***3.47*** | ***4.76*** | ***< 0.01*** |
| LgWF | 4.08 | 0.72 | 4.11 | 0.78 | 0.20 | 0.84 |
| LgCD | 2.58 | 0.63 | 2.57 | 0.69 | 0.03 | 0.98 |
| zipf UK | 4.12 | 0.69 | 4.13 | 0.73 | 0.10 | 0.92 |
| zipf BNC | 4.01 | 0.68 | 4.06 | 0.71 | 0.46 | 0.65 |
| LgHAL | 8.69 | 1.55 | 8.92 | 1.54 | 0.92 | 0.36 |
| OLD | 1.42 | 0.25 | 1.39 | 0.27 | 0.89 | 0.38 |
| ***OLDF*** | ***8.04*** | ***0.35*** | ***8.84*** | ***0.27*** | ***15.65*** | ***< 0.01*** |
| PLD | 1.19 | 0.25 | 1.15 | 0.24 | 0.95 | 0.34 |
| ***PLDF*** | ***8.02*** | ***0.43*** | ***9.31*** | ***0.47*** | ***17.48*** | ***< 0.01*** |
| NPhon | 3.32 | 0.47 | 3.28 | 0.45 | 0.53 | 0.60 |
| NSyll | 1.00 | 0.00 | 1.00 | 0.00 | N/A | N/A |
| NLet | 3.93 | 0.58 | 3.84 | 0.64 | 0.94 | 0.35 |
| AoA | 3.91 | 2.98 | 4.04 | 3.13 | 0.27 | 0.79 |
| Cnc.M | 4.01 | 0.79 | 3.94 | 0.97 | 0.50 | 0.62 |
| Known | 0.99 | 0.03 | 0.99 | 0.03 | 0.10 | 0.92 |
| WNPL | 9.89 | 1.85 | 10.05 | 1.69 | 0.52 | 0.60 |

Low Frequency Neighborhood words: balm, blob, chief, chop, cod, coil, cop, crab, cub, cult, cup, dock, drab, draw, elk, flap, flash, flow, grub, gum, hinge, hooch, horn, hunt, hurt, keep, lab, lamb, lamp, leaf, left, limp, loan, luck, miss, mock, nail, noise, peak, piece, pile, ping, pink, pop, puff, pulp, push, quick, ram, ramp, rap, rough, rub, rug, rush, sash, sham, shift, sick, sink, slam, slew, slip, slot, sock, stone, tag, tang, taps, tray, tub, tug, veil, week, wrap

High Frequency Neighborhood words: bag, band, bar, bat, bead, bean, bet, bib, bin, bum, bun, cake, cap, cent, cite, con, cot, dam, date, dead, dean, dig, dime, dorm, dot, dry, dud, fare, farm, feat, fed, few, fight, fist, fold, ford, fore, fort, forth, fret, fun, gem, grey, heat, heel, helm, help, hem, hide, hind, hive, hold, kid, knit, known, mace, maid, mead, moan, month, nest, norm, raid, sand, sight, theme, thong, toad, vest, wise, worm, worth, yeast, youth, zest

Table A2: Descriptive properties of the stimuli used in Experiments 3 and 4.

|  | Some | | None | |  |  |
| --- | --- | --- | --- | --- | --- | --- |
|  | *M* | *SD* | *M* | *SD* | *t* | *p* |
| CELEX | 4.28 | 0.69 | 4.45 | 0.72 | 1.34 | 0.18 |
| Orth | 7.17 | 3.84 | 6.75 | 3.89 | 0.62 | 0.54 |
| OrthZ | 0.02 | 0.71 | -0.07 | 0.72 | 0.70 | 0.48 |
| OrthF | 19.79 | 24.26 | 15.85 | 24.35 | 0.91 | 0.36 |
| C2 | 115.90 | 76.23 | 119.75 | 80.12 | 0.28 | 0.78 |
| C2Z | -0.03 | 0.80 | 0.06 | 0.79 | 0.67 | 0.51 |
| U2 | 9596.30 | 5515.58 | 8053.83 | 4745.35 | 1.68 | 0.09 |
| ***U2Z*** | ***0.14*** | ***1.12*** | ***-0.20*** | ***0.84*** | ***1.94*** | ***0.05*** |
| ***NHFN*** | ***1.52*** | ***0.80*** | ***0.00*** | ***0.00*** | ***15.11*** | ***<0.01*** |
| LgWF | 4.25 | 0.78 | 4.36 | 0.84 | 0.80 | 0.42 |
| LgCD | 2.72 | 0.68 | 2.77 | 0.70 | 0.45 | 0.65 |
| zipf UK | 4.29 | 0.78 | 4.34 | 0.86 | 0.37 | 0.71 |
| zipf BNC | 4.24 | 0.73 | 4.37 | 0.76 | 0.97 | 0.33 |
| LgHAL | 9.09 | 1.67 | 9.26 | 1.77 | 0.54 | 0.59 |
| OLD | 1.57 | 0.21 | 1.58 | 0.24 | 0.29 | 0.77 |
| OLDF | 7.99 | 0.41 | 7.94 | 0.49 | 0.68 | 0.50 |
| PLD | 1.30 | 0.29 | 1.35 | 0.32 | 0.90 | 0.37 |
| PLDF | 8.14 | 0.75 | 8.21 | 0.85 | 0.47 | 0.64 |
| NPhon | 3.60 | 0.64 | 3.65 | 0.63 | 0.42 | 0.67 |
| NSyll | 1.11 | 0.32 | 1.14 | 0.35 | 0.53 | 0.60 |
| NLet | 4.37 | 0.49 | 4.37 | 0.49 | 0.00 | 1.00 |
| AoA | 4.10 | 2.95 | 3.33 | 2.44 | 1.58 | 0.12 |
| Cnc.M | 3.96 | 0.82 | 3.98 | 0.90 | 0.15 | 0.88 |
| Known | 0.99 | 0.02 | 0.99 | 0.01 | 0.02 | 0.99 |
| WNPL | 9.89 | 1.85 | 10.05 | 1.69 | 0.52 | 0.60 |

Words with no higher frequency neighbors: arms, axis, baker, best, bingo, bird, blue, blues, boat, bomb, bowl, broom, brush, burst, call, chap, chunk, cool, corps, crate, crisp, cuff, cult, czar, damn, dolly, door, drill, duke, face, fatty, felt, find, forty, garb, give, hair, hawk, head, hoax, jump, lava, lily, look, north, plot, porch, push, rise, rock, role, salt, scrap, sick, silly, soap, stock, suit, swirl, swirl, trade, tribe, trot

Words with higher frequency neighbors: apex, boxer, brass, candy, cling, clock, crush, crust, curb, days, fairy, fire, five, gang, goal, golf, grill, jelly, kick, land, limb, loan, meet, mind, monk, opal, pick, pier, plush, plush, pond, poor, rally, rate, ring, rogue, shape, shed, shine, slash, slick, slug, smog, snack, snap, soul, soup, speed, spot, spur, star, step, swamp, tail, take, term, trap, tray, twit, verge, wash, word, works,

References

Balota, D. A., Yap, M. J., Cortese, M. J., Hutchison, K. A., Kessler, B., Loftis, B., & Treiman, R. (2007). The English Lexicon Project. *Behavior Research Methods, 39*, 445-459. https://doi.org/10.3758/ BF03193014

Brysbaert, M, Warriner, A. B., & Kuperman, V. (2014). Concreteness ratings for 40 thousand generally known English word lemmas. *Behavior Research Methods, 46*, 904-911. https://doi.org/10.3758/s13428-013-0403-5

Brysbaert, M., & Biemiller, A. (2017). Test-based age-of-acquisition norms for 44 thousand English word meanings. *Behavior Research Methods, 49*, 1520-1523. https://doi.org/10.3758/s13428-016-0811-4

Brysbaert, M., & New, B. (2009). Moving beyond Kučera and Francis: A critical evaluation of current word frequency norms and the introduction of a new and improved word frequency measure for American English. *Behavior Research Methods, 41*, 977-900. https://doi.org/10.3758/BRM.41.4.977

Ensor, T. M., MacMillan, M. B., Neath, I., & Surprenant, A. M. (2021). Calculating semantic relatedness of lists of nouns using WordNet path length. *Behavior Research Methods, 53*(6), 2430-2438. https://doi.org/10.3758/s13428-021-01570-0

Medler, D. A., & Binder, J. R. (2005). MCWord: An on-line orthographic database of the English language. Madison, WI: Medical College of Wisconsin, Language Imaging Laboratory. Retrieved from www.neuro.mcw.edu/mcword/

Storkel, H. L. (2004). Methods for minimizing the confounding effects of word length in the analysis of phonotactic probability and neighborhood density. *Journal of Speech, Language, and Hearing Research, 47*, 1454-1468. https://doi.org/10.1044/1092- 4388(2004/108)

van Heuven, W. B., Mandera, P., Keuleers, E., & Brysbaert, M. (2014). SUBTLEX-UK: A new and improved word frequency database for British English. *Quarterly Journal of Experimental Psychology, 67*, 1176-1190. https://doi.org/10.1080/17470218.2013.850521

Yarkoni, T., Balota, D., & Yap, M. (2008). Moving beyond Coltheart’s N: A new measure of orthographic similarity. *Psychonomic Bulletin & Review, 15*(5), 971-979. <https://doi.org/10.3758/PBR.15.5.971>
